# Glutathione Transferase as a Potential Marker for Gut Epithelial Injury versus the Protective Role of Breast Milk sIgA in Infants with Rota Virus Gastroenteritis

**DOI:** 10.3889/oamjms.2015.125

**Published:** 2015-11-26

**Authors:** Lobna S. Sherif, Randaa K. Abdel Raouf, Rokaya M. El Sayede, Amany S. El Wakkadd, Ashraf R. Shoaib, Hanan M. Ali, Amira S. El Refay

**Affiliations:** 1 *National Research Centre, Department of Child Health, Giza, Egypt*; 2 *Institute of Postgraduate Childhood Studies - Department of Medical Studies, Cairo, Egypt*; 3 *Kasralainy Cairo University - Pediatric, Cairo, Egypt*; 4 *National Research Centre, Medical Physiology, Cairo, Egypt*; 5 *National Research Centre, Department of Virology, Giza, Egypt*; 6 *National Research Centre, Department of Cell Biology, Giza, Egypt*

**Keywords:** viral gastroenteritis, Rota virus, glutathione transferase, secretory IgA, infants

## Abstract

**BACKGROUND:**

Secretory immunoglobulin A (SIgA) plays an important protective role in the recognition and clearance of enteric pathogens.

**AIM:**

This study was designed to assess if mucosal integrity “measured by secretory IgA (SIgA)” is a protective factor from more epithelial alteration “measured by glutathione transferase” in infants with Rota gastroenteritis and its relation to infants’ feeding pattern.

**PATIENTS AND METHODS:**

This study was conducted on 79 infants aged 6 months and less from those diagnosed as having gastroenteritis and admitted to Gastroenteritis Department in Abo El Rish Pediatric Hospital, Cairo University. Plasma glutathione s-transferases and Stool SIgA were measured using ELISA technique. Rota virus detection was done by Reverse transcriptase PCR.

**RESULTS:**

SIgA was found to be significantly positive in exclusive breast fed infants, Glutathione transferase was significantly more frequently positive in Rota positive cases than Rota negative cases by Reverse transcriptase PCR. A significant negative correlation between Glutathione transferase and Secretory IgA was found, (p < 0.05).

**CONCLUSION:**

Breast feeding should be encouraged and highly recommended in the first two years of life as it provides Secretory IgA to breast fed infants who in turn protect them against epithelial damage caused by Rota viral gastroenteritis.

## Introduction

Rotaviruses are responsible for significant gastrointestinal disease, primarily in children less than 5 years of age worldwide causing massive morbidity and mortality burden especially in the developing countries [1]. Rotavirus infection alters the function of the small intestinal epithelium, resulting in diarrhea with malabsorption secondary to enterocyte destruction [2]. Some asymptomatic rotavirus infections have been reported across all age groups which suggest that both viral and host factors can affect disease severity.

The mucosal immune system constitutes an important first-line defense that protects mucosal surface of the gastrointestinal tract from invasion by potentially pathogenic microbes [3, 4].

The epithelium is a first responder of this mucosal immune system [5] as it creates a tight barrier that separates luminal antigens and gut microbiota from invading the host [6]. The main humoral mediators of this first-line immune system are secretory immunoglobulin A (SIgA) [7, 8]. SIgA plays an important protective role in the recognition and clearance of enteric pathogens [9]. It inhibits colonization and invasion by pathogens and may even inactivate viruses (e.g. rotavirus and influenza virus) inside the secretory epithelial cells and carry the pathogens and their products back to the lumen, thus avoiding cytolytic damage to the epithelium [10].

The glutathione s-transferases (GSTs) are involved in cell protection, antioxidation and detoxification of a range of toxic and foreign compounds within the cell by conjugating them to glutathione. Glutathione s-transferases are predominantly present in liver, kidney and intestine and have been proposed as a potential marker for, amongst others, intestinal epithelial cell damage [11]. Kelly et al., 2004 demonstrated that the glutathione detoxifying system is important in maintaining intestinal barrier integrity [12].

Although intestinal barrier function tests have been improved over the past decades and new tests have emerged, evaluation of intestinal barrier integrity and barrier function loss remains a challenge to both clinicians and scientists.

This study was designed to assess if mucosal integrity “measured by secretory IgA (SIgA)” is a protective factor from more epithelial alteration “measured by glutathione transferase” in infants with Rota gastroenteritis and its relation to infants’ feeding pattern.

## Subjects and Methods

The present study was conducted on 79 infants aged 6 months and less from those diagnosed as having gastroenteritis and admitted to gastroenteritis department in Abo El Rish Pediatric Hospital, Cairo University from November 2011 to September 2012.

The study followed the regulations of the medical ethical committee of the National Research Centre and the ethical committee of Postgraduate Childhood Studies, Ain Shams University. Signed informed consent was collected from mothers of the infants enrolled in the study prior to participation. Self-designed questionnaire was verbally administered to mothers including (age of the infant, breast feeding and weaning practice).

Infants participated in the study were classified into 2 groups; the exclusive breastfed group and the formula feeding group. Exclusive breast feeding was defined as the infant receives only breast milk. No other liquids or solids were given – not even water – with the exception of oral rehydration solution, or drops/syrups of vitamins, minerals or medicines (WHO, 2011) [13]. Formula feeding group included infants who failed to continue breast feeding in the first month or whom follows formula feeding from the first day.

Gastroenteritis was defined if the child experienced ≥ 3 watery or looser-than normal stools and/or forceful vomiting within any 24 hours period within the prior 3 days (WHO, 2009) [14]. The clinical picture and dehydration degree of each infant was assessed based on a direct examination and categorized into mild, moderate and severe using a clinical scoring system [15]. Infants who received Rota vaccine or infants had a history suspecting surgical or extra intestinal causes of diarrhea or receiving immunosuppressive therapy were excluded from the study. Each infant was followed up closely up to discharge to find out the outcome and to record any complications.

### Laboratory investigations

Stool samples were collected using a wooden tongue depressor from the diaper of the infants in 2 sterile plastic cups. 1st cup was used for virus detection and the 2nd was used for estimation of secretory IgA, the 2 cups were saved in -70°C.

The 1st cup: Collected samples were diluted and prepared for RT-PCR for detection of RNA viral gastroenteritis agents in the collected samples. Viral genomes were extracted from 10% diluted stool samples and prepared for detection of viral gastroenteritis by subjection to extraction of both viral RNA and DNA in the samples using Axygen® Kit (Axygen biosciences, Cat. No AP-MN-BF-VNA-250) to facilitate the detection of the RNA Rotavirus [16].

The 2nd cup: Secretory IgA level was determined using commercial kits (Kit no K 8870 immundiagnostik) by ELISA technique intended for the quantitative determination of secretory IgA in stool [17].

Blood Sample was collected using an anticoagulant and centrifuged at 700-1,000 x g for 10 minutes at 4°C. Plasma was separated and stored freezed at -80°C. Glutathione transferase level was estimated by ELISA [18].

### Statistical analysis

Statistical analysis was performed using the SPSS statistical package software for windows version 21 (SSPS Inc, Pennsylvania, USA). Parametric variables are expressed as the mean ± SD. Differences between parametric variables among the obese and overweight versus control groups were evaluated using 2-tailed unpaired t-test. Pearson’s correlation coefficients were used to evaluate correlations between the data exhibiting parametric distribution. P value < 0.05 was considered significant difference and p < 0.005 was considered highly significant difference.

## Results

The descriptive data, feeding pattern and degree of dehydration of the studied infants was shown in Table 1. The mean age of the studied groups was 4.8±1.4 months, 34 infants (43.0%) were female and 45 infants (57%) were males.

**Table 1:** Descriptive data, feeding pattern and degree of dehydration of the studied infants

	Mean ± SD	Range
Age (months)	4.8 ± 1.4	2.0–6.0
Food starting age (months) (N=34)	4.0 ± 0.7	3.0–6.0
	No.	%
Sex
• Male	45	57.0
• Female	34	43.0
Feeding pattern
• Breast	34	43.0
• Formula	45	57.0
Dehydration degree
• Moderate	43.0	57.0
• Severe	34	43.0

According to feeding pattern, 34 infants (43%) were exclusively breast-fed and 45 infants were bottle fed (57%).

As regards the degree of dehydration, 45 infants (57%) suffered from moderate degree of dehydration while 34 infants (43%) suffered from severe degree of dehydration, as shown in Table 1.

Rota virus was found positive in 42 stool samples with a prevalence percentage of 53%, out of them, 18 infants in the exclusive breast feeding group and 24 infants in bottle fed group.

As regards the secretory IgA, it was found to be positive in 25 cases (31.6%) with range (0.0-5637 Ug/ml. In exclusively breast fed infants, secretory IgA was found positive in 24 infants with range (0.0-5637) g/ml and Median (IQR) 652.0 g/ml. However, in bottle fed infants secretory IgA was positive only in 1 infants with range (0.0-0.0) g/ml and Median IQR (0.0) with significant difference (p =< 0.001).

Table 2 shows the effect of SIgA positive and negative on the clinical picture of studied cases. SIgA positive cases showed significantly lower frequency of vomiting than SIgA negative cases, (p < 0.05).

**Table 2:** The effect of SIgA positive and negative on clinical picture

	Positive SIgA (N = 25)	Negative SIgA (N = 54)	χ^2&^	p
Fever	22 (88.0%)	47 (87.0%)	0.014	0.905
Vomiting	20 (80.0%)	51 (94.4%)	3.918	0.048*
General condition				
• Fair	8 (32.0%)	11 (20.4%)
• Moderate	7 (28.0%)	18 (33.3%)	1.267	0.531
• Bad	10 (40.0%)	25 (46.3%)		
Dehydration degree				
• Moderate	15 (60.0%)	30 (55.6%)	0.138	0.711
• Severe	10 (40.0%)	24 (44.4%)		

χ^2&^ Chi square test

* Significant

As regards the plasma glutathione transferase, it was found to be positive in 61 (77%) of cases with range 0.0-41.7 nmol/min/ml and median (IQR) 3.9 ± 1 (1.0-8.0) nmol/min/ml.

Glutathione transferase was found to be positive in 37 cases of Rota gastroenteritis (60%) and negative in only 5 cases (27.8%), (p < 0.05) as shown in Table (3).

**Table 3:** Glutathione transferase in Rota positive cases of the studied infants

	Glutathione transferase positive (N = 61)	Glutathione transferase negative (N = 18)	χ^2&^	p
Rota (RT-PCR)	37 (60.7%)	5 (27.8%)	6.034^&^	0.014

χ^2&^ Chi square test

* Significant

**Figure 1:**
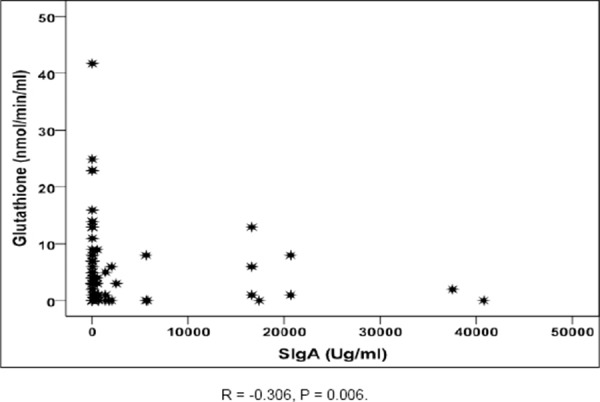
*Correlation between Glutathione transferase level and SIgA level*.

Moreover, Glutathione transferase showed a significant negative correlation with secretory IgA (r= -0.306, P = 0.006) as shown in Figure 1.

## Discussion

Protection at the mucosal surfaces is a very intricate process, which depends on a complex barrier of non-cellular components and the integral structure of the common mucosal immune system [19].

One of the major protective immune mechanisms for the intestinal tract is the synthesis and secretion of IgA. The intestine contains more than 70% of the IgA-producing cells in the body [20]. The next line of defence is the epithelial cell, providing the main physical barrier between the lumen and the host. The IgA antibody traverses the epithelial cell to the lumen by means of a protein carrier (the secretory component) that not only transports the IgA but also protects it against the intracellular lysosomes. IgA does not activate complement and does not enhance cell mediated opsonization or destruction of infectious organisms or antigens.

In humans, the production and functions of sIgA are not optimum until four years of age, however breast milk is able to compensate this when it confers sIgA passively to the suckling infant, providing a robust local immunity. sIgA in the breast milk, mainly colostrum, is a developmental bridge until the infant’s intestines secrete its own [21]. This is in favor to the already stated theory that the only source of secretory IgA is the breast milk.

The profile of SIgA production in breast milk is found to be influenced by maternal age, mood variables, immunological and infectious factors, serum proinflammatory and proimmune cytokines and cortisol levels. Older and stressed women showed lower sIgA while positive life events were correlated with higher breast milk sIgA levels [22] which can explain the absence of sIgA in some breast fed infants in the current work.

In the present study, vomiting symptoms was significantly less frequent in secretory IgA positive cases (P < 0.05). This is can be explained by the inhibitory function of secretory IgA against bacterial and viral attachment to the mucosal epithelium and agglutinates antigens. It is viewed as a benign antibody which fails to bind complement and in turn, unable to elicit an inflammatory response [20].

The glutathione s-transferases (GSTs) are involved in cell protection, antioxidation and detoxification of a range of toxic and foreign compounds within the cell by conjugating them to glutathione. αGST is predominantly present in liver, kidney and intestine and has been proposed as a potential marker for, amongst others, intestinal epithelial cell damage [23].

Kelly et al. 2004 [12] demonstrated that the glutathione detoxifying system is important in maintaining intestinal barrier integrity.

In the present study Glutathione transferase was significantly more frequently positive in Rota positive cases than Rota negative cases by PCR. This is confirming the study theory that Rota virus can cause epithelial erosion documented by glutathione transferase presence in Rota positive cases.

In addition the current study reported a significant negative correlation between Glutathione transferase and Secretory IgA which confirming the put forward theory that good mucosal immunity not only protect against gastroenteritis but even in case of infection it protect against more epithelial damage.

Most important to this end, Secretory IgA inhibits colonization and invasion by pathogens, and pIg R-transported pIgA and pentameric IgM antibodies may even inactivate viruses (e.g. rotavirus and influenza virus) inside of secretory epithelial cells and carry the pathogens and their products back to the lumen, thus avoiding cytolytic damage to the epithelium [24].

This is in favour to our results as in the present study glutathione transferase was significantly lower in Secretory IgA positive cases than Secretory IgA negative cases with P value 0.006 confirming that the presence of Secretory IgA in the intestine of the infant is protecting against epithelial injury. Thus, in addition to the remarkable reinforcement of mucosal defense provided by maternal Secretory IgA and SIgM antibodies as a natural immunological ‘substitution therapy’, it is important to emphasize the positive effects that breast-feeding may exert on immune development including its nutritional value [7].

Because infant’s intestine has a low level of mucus and secretory IgA, the lactating mammary gland is an integral part of the common mucosal immune system [21]. As after parturition, the mother-infant dyad switches from aseptic transfer of nutrients through the umbilicus to dependence on milk and the neonatal intestine to transfer nutrients and protect against enteric pathogens. A rich cornucopia of protective agents in the infant gut and in human milk may compensate for the naive state of adaptive immunity in the neonate [25].

In conclusion, breast feeding is very important in the first two years of life It should be encouraged and highly recommended in the first two years of life as it provides Secretory IgA to breast fed infants which in turn protect them against epithelial damage caused by Rota viral gastroenteritis.
